# Faecal tumour M2 pyruvate kinase: a new, sensitive screening tool for colorectal cancer

**DOI:** 10.1038/sj.bjc.6602033

**Published:** 2004-07-13

**Authors:** P D Hardt, S Mazurek, M Toepler, P Schlierbach, R G Bretzel, E Eigenbrodt, H U Kloer

**Affiliations:** 1 Third Medical Department and Policlinic, Giessen University Hospital, Justus-Liebig-University of Giessen, Rodthohl 6, Giessen 35392, Germany; 2 Institute of Biochemistry and Endocrinology, Veterinary Faculty, University of Giessen, Frankfurter Strasse 100, Giessen 35392, Germany; 3 Medical Department and Surgical Department of the Asklepios Clinic, Goethestrasse 4, Lich 35423, Germany

**Keywords:** tumour M2-PK, colorectal cancer, stool, tumour screening

## Abstract

Proliferating cells, especially tumour cells, express a special isoenzyme of pyruvate kinase, termed M2-PK, which can occur in a tetrameric form with a high affinity to its substrate, phosphoenolpyruvate (PEP), and in a dimeric form with a low PEP affinity. In tumour cells, the dimeric form is usually predominant and is therefore termed Tumour M2-PK. The levels of Tumour M2-PK within tumours and in EDTA-plasma correlate with staging and the ability of the tumour cells to metastasise. Since most colorectal tumours grow intraluminally, it appeared interesting to determine whether Tumour M2-PK is detectable in the faeces of tumour patients. Stool samples were tested by ELISA from controls without colorectal cancer and colorectal cancer patients. Whereas Tumour M2-PK levels were low in the control group (mean value±s.e.m.: 3.3±0.4, *n*=144), they were high in the case of colorectal cancer (56.1±15.3, *n*=60). At a cutoff value of 4 U ml^−1^, the sensitivity was 73%. TNM and Dukes' classification of the tumours revealed a strong correlation between faecal Tumour M2-PK levels and staging. The determination of Tumour M2-PK in faeces provides a new promising screening tool for colorectal tumours.

One alteration consistently found during tumour formation, including gastrointestinal tumours, is the upregulation of glycolytic enzymes. This upregulation takes place at the RNA and protein level, as well as at the level of enzymatic activities (Durany *et al*, 1997; Eigenbrodt *et al*, 1997; Mazurek *et al*, 2000; Hegde *et al*, 2001; Atsumi *et al*, 2002; Birkenkamp-Demtroder *et al*, 2002; Williams *et al*, 2003). In addition, in the case of the glycolytic enzyme pyruvate kinase, a loss of the tissue-specific isoenzymes (L-PK in the liver, M1-PK in muscle and brain and R-PK in erythrocytes) and expression of the pyruvate kinase isoenzyme type M2 (M2-PK) is described in all tumours investigated thus far (Figure 1A

**Figure 1:**
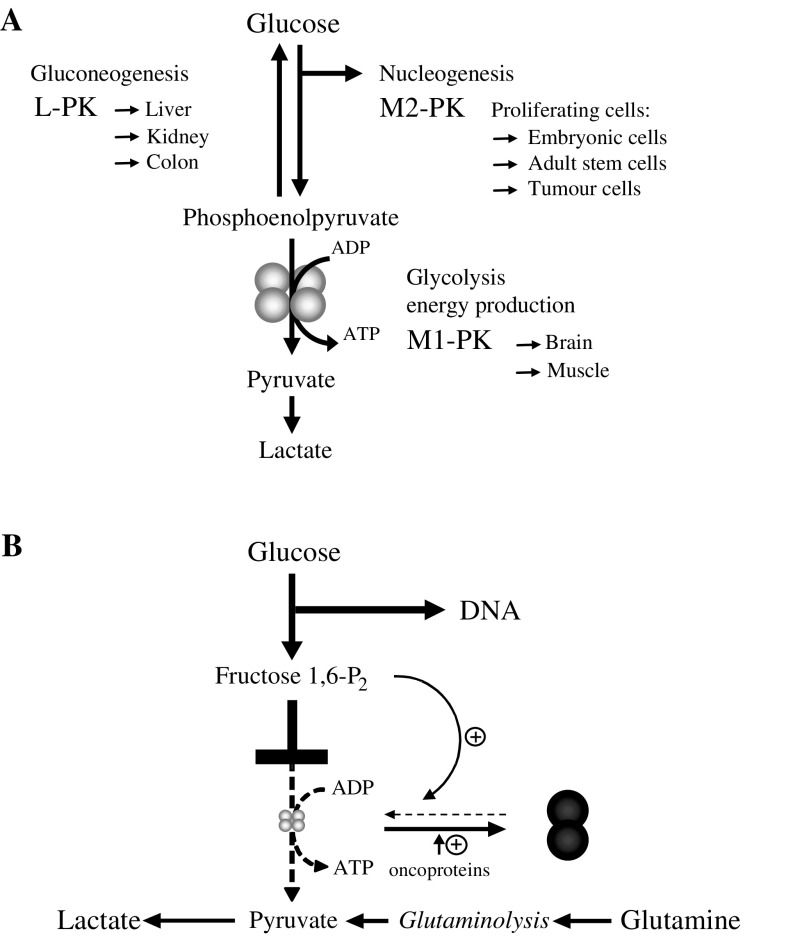
(**A**) Pyruvate kinase isoenzyme equipment of different tissues. (**B**) Metabolic consequences of the dimerisation of M2-PK.

) (Staal and Rijksen, 1991; Mazurek *et al*, 1997; Steinberg *et al*, 1999). The increase in M2-PK levels has been recorded by the determination of enzymatic activities, cellulose acetate electrophoresis, Western blotting and immunohistology (Reinacher and Eigenbrodt, 1981; Staal and Rijksen, 1991; Eigenbrodt *et al*, 1992; Mazurek *et al*, 2000). In healthy tissues, all isoenzymes of pyruvate kinase consist of four subunits whereby hybrids of the different forms can also occur (Saheki *et al*, 1978, 1979). Whereas in the epithelium of the upper gastrointestinal tract (oesophagus and stomach) hybrids between M1 and M2 have been found, the lower gastrointestinal tract (jejunum, colon and rectum) is characterised by hybrids of L and M2-PK (Saheki *et al*, 1978, 1979). In gastrointestinal tumours, only subunits of the type M2 are detectable and pyruvate kinase is mainly in the dimeric form (Mazurek *et al*, 2000). Therefore, the dimeric form of M2-PK has been termed *Tumour M2-PK*. The tetramer : dimer ratio of M2-PK can be quantified by gel permeation, isoelectric focusing or by ELISA (Cerwenka *et al*, 1999; Schulze, 2000; Mazurek *et al*, 1997, 2001; Mazurek and Eigenbrodt, 2003). The upregulation of the M2-PK protein is under the control of HIF-1 and ras, which are both consistently altered in gastrointestinal tumours (Kress *et al*, 1998; Mazurek *et al*, 2001; Nishikawa *et al*, 2002; Mazurek and Eigenbrodt, 2003). The tetramer : dimer ratio of M2-PK is under the control of several oncoproteins, such as pp60^v−src^ kinase, HPV-16 E7 and A-Raf (Figure 1B) (Eigenbrodt *et al*, 1998b; Zwerschke *et al*, 1999; Le Mellay *et al*, 2002). Interestingly, pp60^c−src^ kinase and A-Raf are consistently altered in gastrointestinal tumours (Bolen *et al*, 1987; Iravani *et al*, 1998; Irby *et al*, 1999; Luckett *et al*, 2000; Dehm *et al*, 2001; Dhillon *et al*, 2003).

Determinations of Tumour M2-PK in EDTA-plasma samples of patients with gastrointestinal tumours revealed an upregulation of Tumour M2-PK in oesophageal, gastric, colonic and rectal carcinomas (Hardt *et al*, 2000; Schulze, 2000; Schneider and Schulze, 2003). Recently, we noticed that Tumour M2-PK can be detected and quantified in the faeces of patients with gastrointestinal cancer (Hardt *et al*, 2003). The present study describes the clinical utility of the determination of Tumour M2-PK in the stool of patients with colorectal cancer and control subjects without endoscopic evidence of colorectal neoplasms.

## MATERIALS AND METHODS

### Study protocol

After obtaining informed consent, patients given appointments for colonoscopy for various reasons were asked to provide one stool sample for measuring faecal Tumour M2-PK. A short standard questionnaire about the patient's history was used to record clinical data. Stool samples of patients with colorectal cancer and patients without pathological findings were tested.

Endoscopies were carried out as standard investigations. Histology was obtained from the routine biopsies and/or from surgery. All data were collected prospectively and recorded on standardised forms. The study protocol was approved by the ethics committee of Giessen University Hospital.

### Measurement of faecal tumour M2-PK concentrations

Tumour M2-PK was measured with a commercially available sandwich ELISA (ScheBo® Biotech AG, Giessen, Germany). For sample preparation, 100 mg of the single, random stool specimen was extracted in 10 ml extraction buffer and diluted 1 : 10 in the sample washing buffer provided. The ELISA plate is coated with a monoclonal antibody against Tumour M2-PK. Tumour M2-PK from stool samples or standards binds to the antibody. A second monoclonal antibody, which is biotinylated, binds to Tumour M2-PK during the next incubation. Both monoclonal antibodies against Tumour M2-PK specifically react with Tumour M2-PK (dimeric form of M2-PK) and do not crossreact with the other isoforms of pyruvate kinase (type L, R, M1 and tetrameric M2-PK). In the next incubation step, a conjugate of peroxidase (POD) and streptavidin binds to the biotin moiety. POD oxidises the substrate 3,3′,5,5′-tetra-methyl benzidine (TMB) and the oxidised form of TMB is determined photometrically at 450 nm.

### Statistical analysis

For the statistical comparison of the nonparametrically distributed data, the Kruskall–Wallis ANOVA test was used (Statistica Version 5.0, StatSoft® Inc., Tulsa, USA).

Sensitivity was calculated as the number of patients with colorectal cancer who tested positive for Tumour M2-PK (true positives) divided by the total number of histologically confirmed tumour patients (true positives plus false negatives), expressed as a percentage (i.e. sensitivity=(number of true positives/number of tumour patients) × 100).

Specificity was calculated as the number of controls who tested negative for Tumour M2-PK (true negatives) divided by the total number of control subjects (true negatives plus false positives), expressed as a percentage (i.e. specificity=(number of true negatives/number of patients without pathological findings at colonoscopy) × 100).

## RESULTS

**Figure 2:**
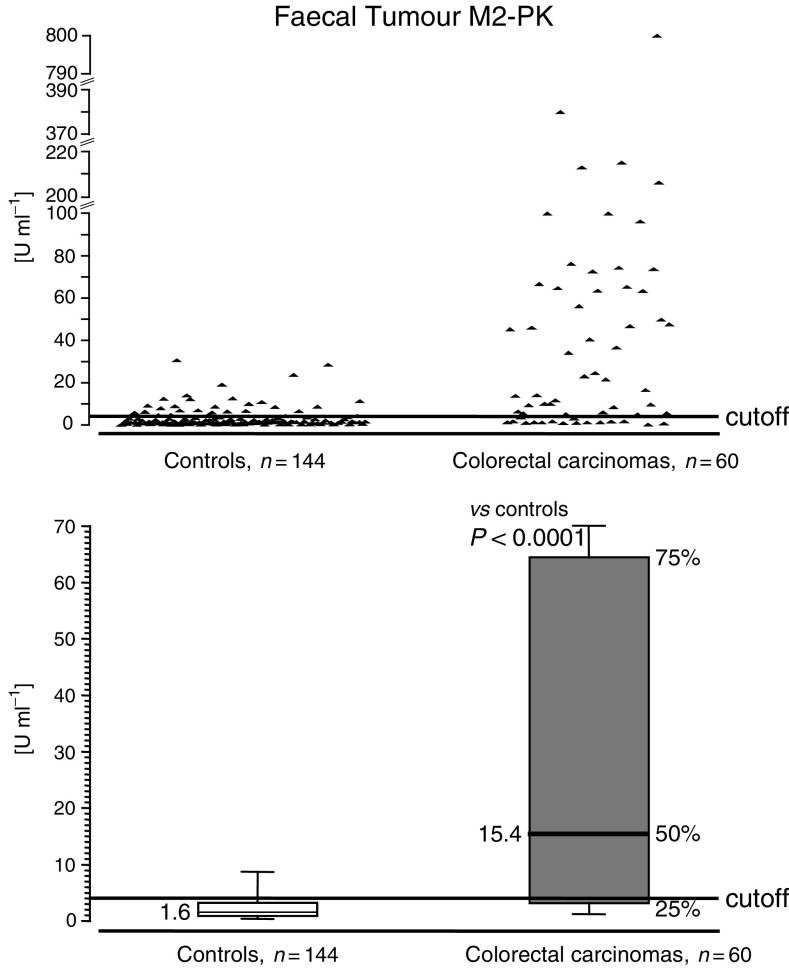
Tumour M2-PK levels in stool samples of patients with colorectal cancer and healthy control individuals. Sensitivity: 73%; specificity: 78%.

In all, 60 patients with colorectal cancer have been evaluated to date. A total of 144 patients underwent total colonoscopy without any pathological findings and served as controls. The range of faecal Tumour M2-PK levels is shown in Figure 2

. There is a highly significant difference between tumour patients and controls. At a cutoff level of 4 U ml^−1^, the sensitivity was calculated to be 73% and the specificity as 78%. TNM classification of the colorectal tumours according to the criteria of the American Joint Committee on Cancer (AJCC, 2002), as well as the Dukes' classification, revealed a strong correlation between the amount of faecal Tumour M2-PK and staging (Table 1

**Table 1:** Correlation between faecal tumour M2-PK levels and staging

**Classification**	***n***	**Median (U ml^−1^)**	**Mean (U ml^−1^)**	**s.e.m. (U ml^−1^)**	**Range (U ml^−1^)**
Controls	144	1.6	3,3	0.4	0.1–31
					
T1	7	5.5	11.1	5.9	1.3–45
T2	11	10.0	23.8	9.9	1.1–100
T3	33	34.2	55.5	13.7	1.0–380
T4	9	47.8	132.7	86.2	0.2–800
					
Dukes' A	17	6.4	19.4	6.9	1.1–100
Dukes' B	16	14.5	31.5	8.5	1.0–100
Dukes' C	17	46.8	57.1	15.9	0.2–215
Dukes' D	10	36.4	156.2	81.1	0.7–800

TNM classification according to AJCC, 2000: T1: tumour invades submucosa; T2: tumour invades muscularis propria; T3: tumour invades through muscularis propria into subserosa or into nonperitonealised pericolic or perirectal tissues; T4: tumour directly invades other organs or structures and/or perforates visceral peritoneum. Dukes' staging according to Cuthbert Dukes, 1932: Dukes' A: the cancer confines to most superficial cell layers of colon and rectum; Dukes' B: the cancer penetrates the colon wall; Dukes' C: the cancer spreads to the lymph nodes; Dukes' D: the cancer spreads to distant organs. *n*=number of individuals; s.e.m.=standard error of the mean.

; Figure 3

**Figure 3:**
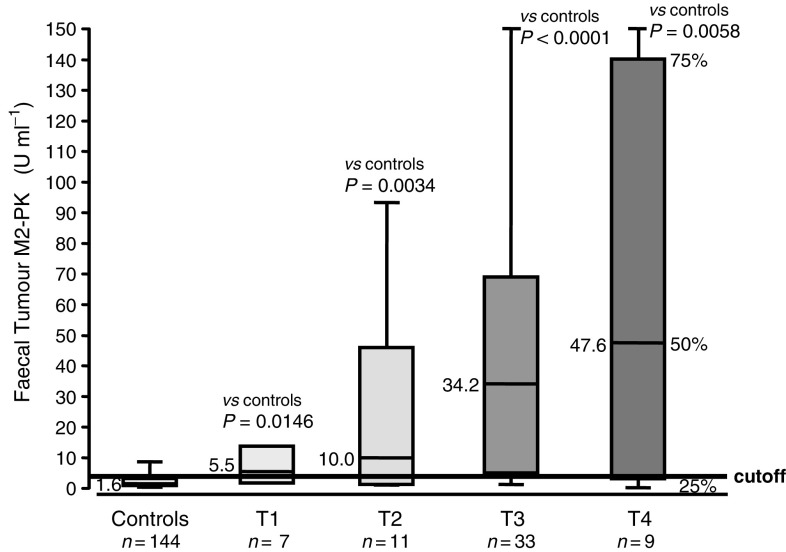
Correlation between faecal Tumour M2-PK levels and TNM staging. Sensitivities: T1, 57%; T2, 64%; T3, 78%; and T4, 78% (see Table 1).

). The sensitivities increased from 57 and 59% in the case of T1 and Dukes' A, respectively, to 78% in the case of T4 and 90% in the case of Dukes' D.

The intra-assay variance was evaluated by 18-fold determination of five samples (5–66 U ml^−1^), giving an average coefficient of variance (CV) of 7.9% (3.5–13.6%). The interassay variance was calculated with five samples between 4 and 73 U ml^−1^, tested on 10 different days. The average CV was 7.3% (3.8–12.6%).

## DISCUSSION

In the present study, Tumour M2-PK was measured in the faeces of patients with colonoscopy-proven cancer of the colon and rectum. The data presented here are the results of the first extensive study in this field, which was conducted in secondary-care gastrointestinal clinics and reflects the current presentation of colorectal cancer in this setting.

The faecal levels of Tumour M2-PK are significantly higher in patients with colorectal cancer than in the control group without colorectal cancer (Figure 2). Overall sensitivity is 73% and specificity is 78%. All patients in our study underwent complete colonoscopy to confirm (together with histology) colorectal cancer or exclude it. When similarly compared against colonoscopy, screening with a guaiac-based faecal occult blood test (FOBT) had a sensitivity of 24% in a recent study (Lieberman *et al*, 2001). Nevertheless, large randomised studies of screening with FOBT have demonstrated a reduction in mortality or risk of 15–33% (Hardcastle *et al*, 1996; Mandel *et al*, 1999; Jorgensen *et al*, 2002).

Owing to the high sensitivity, the determination of Tumour M2-PK in stool samples might be a valuable new screening tool for colorectal cancer. Furthermore, a close correlation was found between Tumour M2-PK levels and tumour staging for both TNM and Dukes' classification (Table 1; Figure 3).

An increase of Tumour M2-PK levels has also been measured in EDTA-plasma samples of patients with colorectal cancer (Hardt *et al*, 2000; Schulze, 2000; Schneider and Schulze, 2003). The sensitivity of the Tumour M2-PK EDTA-plasma test was lower (nonmetastasising cancer: 48%; metastasising cancer: 54%) than the Tumour M2-PK stool test (Dukes' A: 59%; Dukes' D: 90%). Furthermore, Tumour M2-PK levels are also increased in EDTA-plasma samples from patients with solid tumours at various sites, including renal, lung and breast cancers (Oremek *et al*, 1999; Wechsel *et al*, 1999; Lüftner *et al*, 2000; Schneider *et al*, 2000; Mazurek *et al*, 2002). Therefore, for the screening of colorectal cancer the determination of faecal Tumour M2-PK is superior to the EDTA-plasma test.

However, analogous to studies in breast, lung and kidney cancer patients (Wechsel *et al*, 1999; Lüftner *et al*, 2000; Hoopmann *et al*, 2002; Schneider *et al*, 2002), a possible field of application for the Tumour M2-PK EDTA-plasma test may be the monitoring and follow-up of patients with colorectal cancer.

Immunohistological staining of Tumour M2-PK in various rat and human tumours (breast, renal, lung, colon, rectal and skin tumours) revealed that increased Tumour M2-PK in tumour cells is a general metabolic alteration during tumorigenesis and correlated with malignancies of the tumours (Reinacher and Eigenbrodt, 1981; Bahnemann *et al*, 1990; Eigenbrodt *et al*, 1992; Schneider *et al*, 2000).

The upregulation of the M2-PK isoenzyme can be caused by mutations in the ras gene and upregulation of HIF-1 (Kress *et al*, 1998; Mazurek *et al*, 2001). HIF-1 is stabilised by p53 mutations (Kress *et al*, 1998; Ravi *et al*, 2000). Indeed, mutations in the ras oncogene and p53 antioncogene are detectable in 40–60% of colorectal cancers (Bos, 1989; Kinzler and Vogelstein, 1996; Dong *et al*, 2001; Rengucci *et al*, 2001; Nishikawa *et al*, 2002; Smith *et al*, 2002).

The determination of mutated oncogenes and antioncogenes in stool samples has the advantage of high specificity. For example, mutations of K-ras, p53 or within the adenomatous polyposis coli (APC) gene were not detected in any stool samples of healthy controls (Rengucci *et al*, 2001; Nishikawa *et al*, 2002; Traverso *et al*, 2002). However, due to the genetic heterogeneity within colorectal cancer, a multitarget assay panel (e.g. mutations of k-ras, p53 and APC genes, as well as Bat-26 (a microsatellite instability marker) and highly amplifiable ‘long’ DNA) has to be screened to reach high sensitivities (63–95%) (Ahlquist *et al*, 2000; Tagore *et al*, 2003).

Since M2-PK is a direct target of several oncoproteins, the determination of faecal Tumour M2-PK might be more sensitive than the detection of mutations in the oncoproteins and antioncoproteins themselves.

In contrast to many other genes, no mutations have yet been found in the M2-PK gene (http://www.pubmed.gov: nucleotide, tumour pyruvate kinase type M2). Furthermore, the overexpression of a mutated M2-PK protein suppresses A-Raf transformation in NIH 3T3 cells, which underlines the central role of M2-PK within the tumour metabolome (Le Mellay *et al*, 2002). Indeed, pyruvate kinase controls the exit of glycolysis and is responsible for net ATP production of this pathway, especially under hypoxic conditions. M2-PK can switch between a highly active tetrameric form with a high affinity to its substrate, phosphoenolpyruvate (PEP), and a nearly inactive dimeric form with a low PEP affinity (Figure 1B). The tetramer : dimer ratio of M2-PK is regulated by oncoproteins and metabolic intermediates, such as fructose 1,6-P2 and serine, and determines whether glucose carbons are converted to pyruvate and lactate under the production of energy (tetrameric form) or are channelled into synthetic processes, such as DNA, phospholipid and amino-acid synthesis (dimeric form) (Mazurek *et al*, 1997; Griffiths *et al*, 2002; Boren *et al*, 2003). When M2-PK is mainly in the nearly inactive dimeric form (Tumour M2-PK) energy is provided by the degradation of the amino-acid glutamine to lactate, which has been termed *glutaminolysis* (Figure 1B) (http://www.metabolic-database.com).

The oscillation between the highly active tetrameric form and the nearly inactive dimeric form allows optimal adaptation of the tumour metabolome to the varying nutrient and oxygen supply constantly found in solid tumours (Eigenbrodt *et al*, 1998a; Vaupel *et al*, 2003).

Tumour M2-PK is detected with an ELISA that can be easily performed in every routine laboratory. Samples are stable and the sensitivity of the Tumour M2-PK test is higher than the detection of mutations in p53, ras or APC (Rengucci *et al*, 2001; Nishikawa *et al*, 2002; Traverso *et al*, 2002). Therefore, the detection of Tumour M2-PK levels in the stool might provide an interesting screening tool for colorectal cancer. To further validate this new test in a larger cohort, a larger cross-sectional study will be performed in the future.
